# Chemical Structural Coherence Principle on Polymers for Better Adhesion

**DOI:** 10.3390/polym14142829

**Published:** 2022-07-12

**Authors:** Alena L. Krapivko, Yegor D. Ryabkov, Fedor V. Drozdov, Nikolay A. Yashtulov, Nikolay K. Zaitsev, Aziz M. Muzafarov

**Affiliations:** 1 M. V. Lomonosov Institute of Fine Chemical Technologies, MIREA—Russian Technological University, Vernadskogo Prospect, 86, 119571 Moscow, Russia; ryabkov_e@mirea.ru (Y.D.R.); yashtulovna@mail.ru (N.A.Y.); 2 N.S. Enikolopov Institute of Synthetic Polymeric Materials, Russian Academy of Sciences, Profsoyuznaya St., 70, 117393 Moscow, Russia; drozdov@ispm.ru (F.V.D.); aziz@ispm.ru (A.M.M.); 3 Econics-Expert Ltd., Akademika Bakuleva St., 6, 117513 Moscow, Russia; nk_zaytsev@mail.ru

**Keywords:** fluoropolymers, fluorinated ORMOSIL, anodized aluminum, adhesion, surface tuning

## Abstract

Composite materials are the most variative type of materials employed in almost every task imaginable. In the present study, a synthesis of a novel perfluoroalkyltriethoxysilane is reported to be used in creating composites with polyhexafluoropropylene—one of the most indifferent and adhesion-lacking polymers existing. The mechanism of adhesion of hexafluoropropylene is proved to be due to chemical structural coherence of perfluoroalkyltriethoxysilane to a link of polyhexafluoropropylene chain. The ability of perfluoroalkyltriethoxysilane to attach to surfaces was studied by FT-IR spectroscopy of modified glass microspheres. Although the perfluoroalkyltriethoxysilane surface modifier allowed partial adhesion of polyhexafluoropropylene, some detachment took place; therefore, the surface nanostructuring was used to increase its specific area by aluminum foil anodizing. An anodized aluminum surface was studied by scanning electron microscopy. The resulting composite consisting of anodized aluminum, perfluoroalkyl surface modifier, and polyhexafluoropropylene layer was proved to be stable, showed no signs of detachment, and is a promising material for usage in harsh environments.

## 1. Introduction

Composite materials of different application gain increasing attention nowadays. Composite materials are employed in various areas, including construction [1,2,3,4,5], aerospace [6], biomedical [7,8,9,10,11], sensor [12,13,14,15,16,17,18,19,20], and many other industries. Composite material is a system consisting of at least two phases with a pronounced interface border. Thus, it is crucial for composite stability that its constituents must have proper adhesion to each other [21]. Without proper adhesion of two heterogenous materials a significant decrease in mechanical strength becomes possible up to rapid destruction of the composite [22,23,24,25,26,27].

There are several methods for increasing adhesion between polymer matrix and fillers. Most of these methods are related to surface modification [28,29]. Nanostructuring is a promising method for increasing specific surface area, chemical structure of the surface and its roughness, and that leads to better adhesion [30,31]. A chemical surface modification can be employed to create “anchor” functional groups on different surfaces, which will allow a polymer to attach to the surface due to chemical structural coherence, and silanes with various architecture frequently serve this purpose [32,33].

Among many materials used as polymer matrices, fluorinated polymers occupy a special place. These materials have outstanding properties, such as durability, photoaging resistance, and chemical inactivity [7,34,35,36,37,38,39] due to high carbon-fluorine bond energy. One of the most stable and promising materials is polyhexafluoropropylene (PHFP) [40]. PHFP is a perfluorinated polymer with the least surface energy and a wide range of working temperatures. However, fluoropolymers have extremely low adhesion to almost all materials due to the abovementioned low surface energy, and that makes them challenging to work with as composite matrices [41,42]. Neither the nanostructuring mentioned above nor the use of known commercially available silanes make it possible to obtain stable composites based on PHFP.

In this paper, we report a method for significantly improving the adhesion of perfluorinated materials using PHFP as an example. In fact, this work is an extension of the gecko-tape idea of Nobel laureate Andre Geim [43], but we decided to go down to the molecular level of coherence. It implies that a structural element of the fluorinated matrix is used as a part of the specially designed surface modifier. This paper describes the direct synthesis of a new perfluoroalkyltriethoxysilane with a dimer of hexafluoropropylene molecular fragment, i.e., with a structural element of PHFP. The modifier could be used for surfaces that need to be attached to the polymer matrix–whether it is a filler particle or a substrate surface. We proposed that although van der Waals interactions between similar structural parts are weak, due to a great number of interactions, the desired effect of PHFP adhesion could be attained. The modifier allows one to attach the fluorinated polymer to the glass or metal surface, and a modifier for epoxy resin could be obtained in case of copolymerization with phenyltriethoxysilane. The well-known aluminum foil anodizing technique [44,45,46,47] was engaged as a method for surface nanostructuring and further increasing of the fluorinated polymer adhesion to metal surface.

## 2. Materials and Methods

### 2.1. Materials

Aluminum foil A5 DPRNM 0.1 × 500 mm State Standard 618-2014 was purchased from RT-Techpriemka (Rostech, Moscow, Russia), analytical grade acids were purchased from Chimmed (Moscow, Russia), and auxiliary electrode was purchased from Econics-Expert (Moscow, Russia). Hexane for syntheses purposes was purchased from Sigma Aldrich (St. Louis, MO, USA), as well as glass microspheres with particle size 1–40 μm, trichlorosilane, carbamide, ethanol, toluene, Carsted catalyst, acetone, and calcium chloride as drying agent. Hexafluorobenzene was provided by P&M Invest (Moscow, Russia). Polyhexafluoropropylene was kindly provided by Laboratory of Chemical Reactions under High Pressure of N. D. Zelinsky Institute of Organic Chemistry Russian Academy of Sciences, Moscow, Russia, the synthesis was reported earlier [48]. 2,2,3,3,4,4,5,5,6,6,6-Undecafluoro-N-(3-methyldiethoxysilylpropyl)hexanamide surface modifier was kindly provided by N.S. Enikolopov Institute of Synthetic Polymeric Materials, Russian Academy of Sciences, Moscow, Russia.

### 2.2. Synthesis of 1,1,1,2,2,3,3-Heptafluoro-4,4-bis(trifluoromethyl)pentyltriethoxysilane

**Figure 1:**
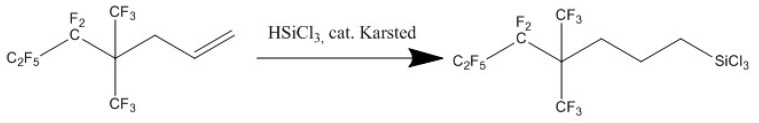
Trichlorosilane addition reaction scheme.

The initial fluorine-containing precursor for this synthesis has been obtained by our group earlier by a known method [49]. Reaction was carried out in argon atmosphere. Trichlorosilane was introduced in reaction flask through a septum to the fluorinated precursor, then Carsted catalyst was added. Reaction mixture was intensively mixed for 24 h in room temperature. Trichlorosilane was then removed from reaction mixture by distillation at 50 °C. Reaction product was then distilled, fraction with boiling point at 94 °C was collected (Figure 1).

**Figure 2:**
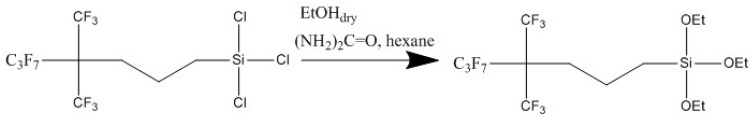
Etoxylation reaction scheme.

A mixture of carbamide, ethanol, and hexane was added to four-necked round bottom flask with mechanical stirring in argon atmosphere. The product of previous stage was introduced to reaction mixture by dropping funnel at low rate and intensive stirring. After full introduction of the trichlorosilane the reaction was heated to 50 °C and was held under argon atmosphere and intensive stirring for 12 h (Figure 2). The resulting product was distilled and fraction with boiling point at 120 °C.

### 2.3. Aluminum Foil Anodizing

The formation of nanopeforations on the aluminum surface was performed on aluminum foil samples with a purity of 98.5% by high-voltage anodizing. Before anodizing, aluminum samples were cleaned with acetone. Amounts of 0.3 M phosphoric acid, 0.3 M oxalic acid, and 0.3 M sulfuric acid were used as the background electrolyte. Oxygen was removed from the solution by purging with 99.999% purity nitrogen, while the gas purging continued during anodization. The auxiliary electrode was a platinum wire with a diameter of 0.5 mm and a length of 20 cm, which was twisted into a spiral with a coil diameter of 1 cm, at a charge of 10 A and a voltage maintenance accuracy of 0.5 V. An aluminum foil sample with an uninsulated area of 4 cm^2^ was placed in a cell equipped with a RITM-01 magnetic stirrer (Econics-Expert, Moscow, Russia), which was a heat resistant glass. The background electrolyte temperature was maintained constant at 21 °C by passing tap water at the same temperature through the cell jacket. The sample treated for the selected time and at the selected voltage was washed with bidistilled water, dried in air for 24 h, and then examined on a Hitachi-SU8200 scanning electron microscope (Hitachi, Tokyo, Japan) in a low-voltage, low-temperature mode at an accelerating voltage of 20 kV and temperature of 77 K. Scanning electron microscopy was the main method for determining the number and size of nanoperforations on the aluminum surface. Anodized aluminum samples preparation is described in Table 1.

The Digimizer program Version 5.7.2 (MedCalc Software Ltd., Ostend, Belgium) was used to process images and calculate average nanoperforation diameters.

### 2.4. Sample Surface Preparation

Anodized aluminum foil samples were degreased in acetone, air-dried, and then submerged in NaOH 2.5 M solution for 30 s with a following rinse in distilled water. The prepared aluminum foils were dried in desiccator over calcium chloride for 24 h.

### 2.5. Sample Surface Modification

The prepared aluminum foils were submerged in 1% solution of surface modifier in hexafluorobenzene for 60 s, then these samples were placed in an oven at 150 °C for an hour. Prepared samples were air cooled.

### 2.6. Polymer Films Application

A 10% solution of polymer in appropriate solvent was prepared. Hexafluorobenzene was used for polyhexafluoropropylene, toluene was used for polymethylmethacrylate, and acetone was used for polytetrafluoroethylene copolymer.

The prepared aluminum foils with surface area of 3 cm^2^ were coated with 500 μL of the prepared solution and air dried for 24 h.

### 2.7. Shear Strength Test Samples Preparation

A 10% solution of polymer in appropriate solvent was prepared. Hexafluorobenzene was used for polyhexafluoropropylene, toluene was used for polymethylmethacrylate, and acetone was used for polytetrafluoroethylene copolymer. 

The prepared aluminum foils with surface area of 3 cm^2^ were coated with 500 μL of the prepared solution, covered with another aluminum foil, and air dried for 24 h.

### 2.8. Shear Strength Test

Shear strength tests were performed on Instron 5942 (Instron, Norwood, MA, USA). ASTM D1002 method was employed—a lap shear test which is performed to determine the shear strength of an adhesive that is applied to two metal plates and pulled to failure.

### 2.9. Glass Microspheres Modification

Glass microspheres surface was prepared the same way as the aluminum foils. Treated glass microspheres were submerged in perfluoroalkyltriethoxysilane solution for 10, 30, 60 and 300 s, and then were placed in an oven at 150 °C for an hour.

### 2.10. 1,1,1,2,2,3,3-Heptafluoro-4,4-bis(trifluoromethyl)pentyltriethoxysilane Characterization

NMR studies were carried out on WM-250 spectrometer (Bruker Corporation, Billerica, MA, USA) in hexafluorobenzene solution.

### 2.11. Surfaces Characterization

#### 2.11.1. Glass Microspheres Surface Characterization

Fourier Transform Infrared Spectroscopy (FT-IR) method was employed to prove glass surface modification on Bruker Equinox 55 FT-IR spectrometer (Billerica, MA, USA).

#### 2.11.2. Aluminum Surface Characterization

Water contact angles on treated aluminum foil surfaces were obtained and measured on Biolin Scientific Theta lite (Gothenburg, Sweden).

## 3. Results and Discussion

### 3.1. 1.1,1,2,2,3,3-Heptafluoro-4,4-bis(trifluoromethyl)pentyltriethoxysilane Characterization

**Figure 3:**
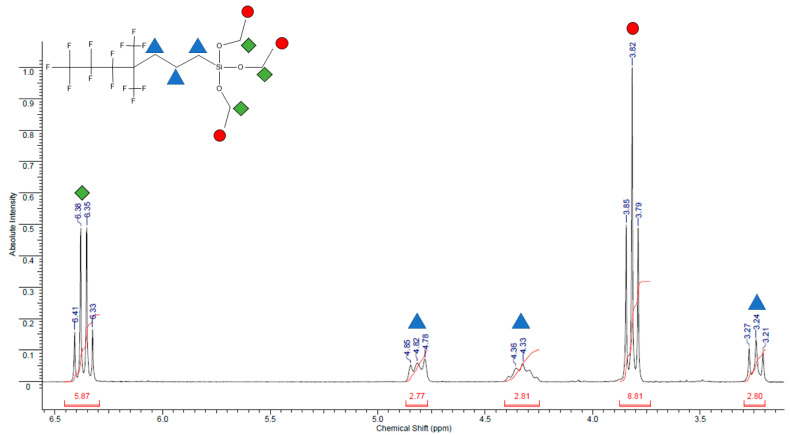
NMR spectrum of 1,1,1,2,2,3,3-heptafluoro-4,4-bis(trifluoromethyl)pentyltriethoxysilane ^1^H NMR (70 MHz, C_6_F_6_), δ, m.d. = 3.24 (t, 2H, J = 8.3 Hz, CH2), 3.82 (t, 9H, J = 7.0 Hz, OCH_2_CH_3_), 4.32–4.36 (m, 2H, CH_2_), 4.82 (t, 2H, J = 8.3 Hz, CH_2_), 6.38 (q, 2H, J = 7.0 Hz, OCH_2_CH_3_).

NMR spectrum of synthesized modifier is shown in Figure 3.

### 3.2. Glass Surface Characterization

**Figure 4:**
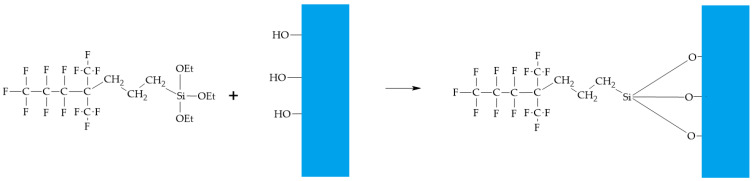
Modifier attachment to hydroxyl-containing surfaces mechanism.

The synthesized 1,1,1,2,2,3,3-heptafluoro-4,4-bis(trifluoromethyl)pentyltriethoxysilane can be attached to hydroxyl-containing surfaces in the same way as other organosilanes [50]. The interaction between the modifier and glass and metal surfaces that were previously treated with sodium hydroxide to create hydroxyl groups was studied. Modifier addition mechanism is shown in Figure 4. Thus, the surface modification can be referred to as chemosorption of the modifier to the hydroxyl-containing surface, creating covalent Si-O-Si bonding.

**Figure 5:**
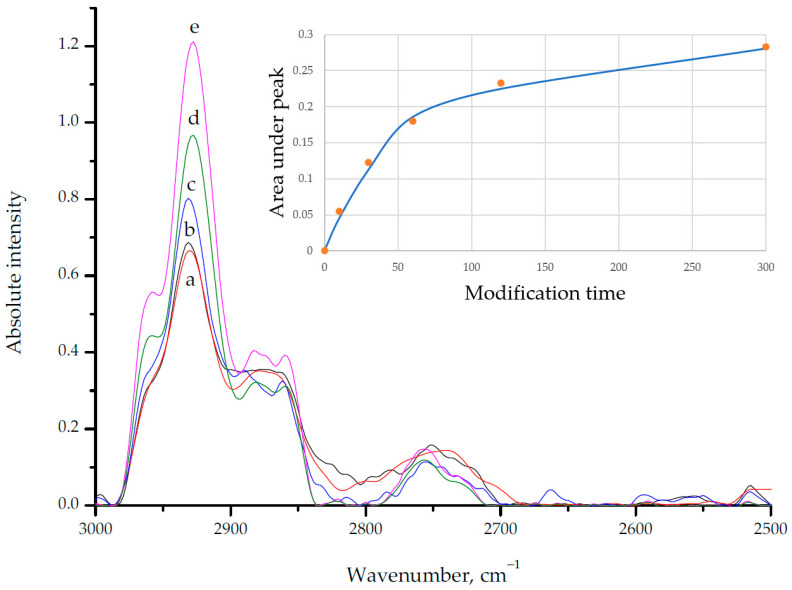
FT-IR spectra of modified glass microspheres with different modification times: a—10 s, b—30 s, c—60 s, d—120 s, e—300 s. Insertion–calculated peak area fitted by Langmuir-type model Equation (1).

The FT-IR spectra of glass microspheres are shown in Figure 5. The adsorption band of 1,1,1,2,2,3,3-heptafluoro-4,4-bis(trifluoromethyl)pentyltriethoxysilane modifier was detected at 2928 cm^−1^ (C–H, stretching).

The kinetics of glass microspheres modification was studied by treating glass microspheres with modifier with varying exposure times (Figure 5). One can see increasing values of the C–H band peak intensity that corresponds to increasing quantity of propyl groups of the modifier. The longer modification time is, the more molecules of modifier absorb on glass surface. The dependence of calculated peak area is well-fitted by the Langmuir-type equation as seen on the Insertion in Figure 5.

(1) $$A=\frac{A_{\max}\tau }{\tau +K}$$

where *A* represents peak area, *A*_max_—maximum peak area, *τ*—modification time, and *K*—equilibrium constant of modifier chemosorption reaction. In this case, *K* represents time needed for half of the chemosorption sites to react. Numerical fitting for the observed reaction yields the values *A*_max_ = 0.33 a.u., *K* = 50.6 s. C–F adsorption band is overlapped by Si–O adsorption band and cannot be used to estimate the modification success.

### 3.3. Anodized Aluminum Characterization

**Figure 6:**
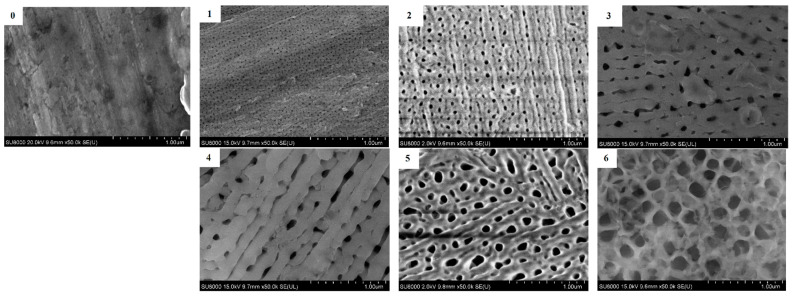
SEM images of anodized aluminum foil samples, anodizing conditions and sample numbers are shown in Table 1. The nanoperforations diameters are: (**0**)—aluminum without anodizing; (**1**)—10 nm; (**2**)—30 nm; (**3**)—50 nm; (**4**)—70 nm; (**5**)—100 nm; (**6**)—150 nm.

According to earlier studies, it is known that aluminum specific surface area obtained by anodizing can be manipulated by changing the technological parameters such as voltage, exposition time, electrolyte composition etc. [51,52]. Anodized aluminum samples 1–6 were prepared by using certain regimes with nanoperforation diameters of 10, 30, 50, 70, 100, and 150 nm, respectively. Figure 6 shows SEM images of these samples’ surfaces, which show the grids of the resulting regular patterns of nanoperforations. Sample № 0 is aluminum that has not been anodized and is shown here for comparison.

### 3.4. Modified Aluminum Surface Characterization

**Figure 7:**
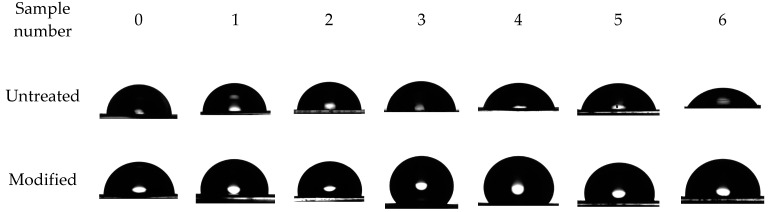
Water contact angles comparison of pictures of modified and untreated anodized aluminum foil samples.

**Figure 8:**
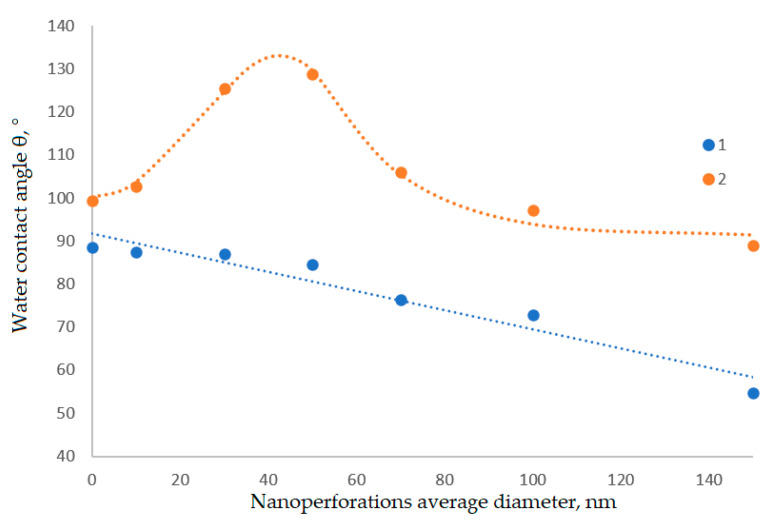
Water contact angle dependence of nanoperforation average diameters, 1—untreated anodized aluminum, 2—modified aluminum.

Data obtained from water contact angle comparison are shown in Figure 7 and Figure 8. It is clear that the dependence of water contact angles to nanoperforation average diameter in non-linear with a maximum somewhere in between 40 nm to 60 nm due to the attainment of maximum specific surface area.

This step is needed to increase the number of adsorption sites for the polymer after the surface modification. Further anodization, as it seems, leads to lower values of specific surface area due to the merging of nanoperforations together and destruction of perforations’ walls with formation of fewer amounts of bigger perforations. Anodized aluminum foils without surface modification show linear decrease in water contact angles due to hydrophilic nature of aluminum, that is further strengthened by increased specific surface area.

### 3.5. Polymer Films

**Figure 9:**
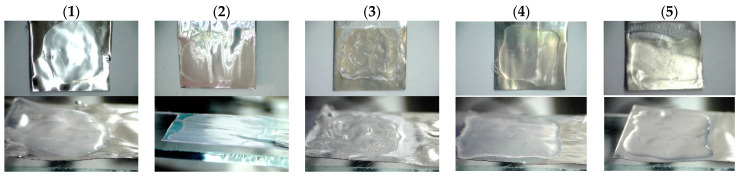
Pictures of polymer films on aluminum samples: (**1**)—polymethylmethacrylate film on untreated aluminum; (**2**)—polymethylmethacrylate film on anodized aluminum; (**3**)—polytetrafluoroethylene copolymer film on anodized aluminum; (**4**)—polyhexafluoropropylene film on anodized aluminum surface; (**5**)—polyhexafluoropropylene film on modified and anodized aluminum surface.

Examples of films of various polymers deposited on untreated and treated aluminum substrates are shown in Figure 9.

Sample 1 shows that polymethylmethacrylate (PMMA) did not attach to untreated aluminum surface at all. Sample 2 shows even attachment of PMMA to anodized aluminum surface. Sample 3 is an unevenly attached layer of PTFE copolymer on anodized aluminum surface. PTFE has significantly lower surface free-energy and it takes additional effort to provide proper adhesion on desirable surfaces. Sample 4 is a PHFP layer that has completely detached from the anodized aluminum surface during the drying process. In contrast, the PHFP film on the modified and anodized aluminum surface adhered very well without any sign of detachment (Sample 5). As can be seen from the pictures, polymers do attach better to nanostructured surfaces than to untreated aluminum due to the lack of adhesion centers of the latter. However, PHFP film did not stick to anodized surface at all due to its extremely low surface energy. PHFP film on the modified and anodized aluminum surface attached completely without any signs of detachment. This is possible due to chemical structural coherence that was attained by treating the anodized aluminum surface with the modifier with an element of PHFP structure. Modification of the surface creates large number of adhesion sites with the PHFP links, acting as crystallization seeds for the polymer. And the more sites the desirable surface has, the better adhesion will be attained due to larger numbers of van der Waals interactions between the PHFP chains and modifier’s fluorinated substituent. To increase the number of these sites we simply increased the specific surface area on aluminum samples by anodizing, and that manipulation ensured the even adhesion of the PHFE to the modified surface.

**Figure 10:**
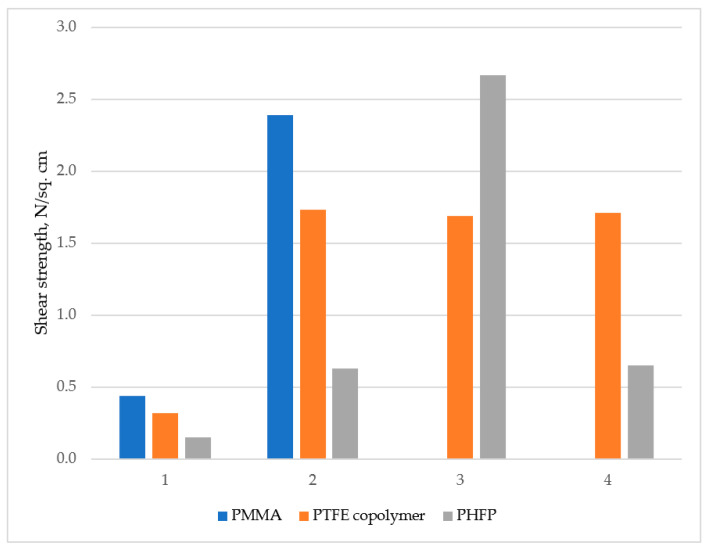
Sheer strength of adhesive systems with abovementioned polymers: (**1**)—untreated aluminum foils, (**2**)—anodized aluminum foils, (**3**)—anodized aluminum foils with 1,1,1,2,2,3,3-heptafluoro-4,4-bis(trifluoromethyl)pentyltriethoxysilane modification, (**4**)—anodized aluminum foils with 2,2,3,3,4,4,5,5,6,6,6-Undecafluoro-N-(3-methyldiethoxysilylpropyl)hexanamide modification.

As can be seen from Figure 10, the shear strength of composite material changes dramatically when materials have structural coherence. The polymer samples on untreated aluminum foil proved to be unstable even at the drying stage. Samples with nanostructured aluminum foils show significant increase in shear strength, although PHFP sample was still unstable. Samples with anodized aluminum foils with 1,1,1,2,2,3,3-heptafluoro-4,4-bis(trifluoromethyl)pentyltriethoxysilane modification show much better adhesion for PHFP, and slight decrease of shear strength for PTFE copolymer. Samples with anodized aluminum foils and 2,2,3,3,4,4,5,5,6,6,6-Undecafluoro-N-(3-methyldiethoxysilylpropyl)hexanamide modification showed almost no adhesion increase for PHFP compared to unmodified anodized aluminum surface. These results make it clear that structural coherence between PHFP and 1,1,1,2,2,3,3-heptafluoro-4,4-bis(trifluoromethyl)pentyltriethoxysilane does in fact takes place and it is this interaction that plays a key role in the demonstrated method for improving adhesion.

## 4. Conclusions

The 1,1,1,2,2,3,3-heptafluoro-4,4-bis(trifluoromethyl)pentyltriethoxysilane modifier was synthesized. The ability of modifier to attach to surfaces containing hydroxyl groups was studied along with the kinetics of the modification and the results implied that the modifier successfully attached to the surfaces forming a covalent chemical bond. The surface modification technique was optimized. The adhesive ability of the modifier towards the PHFP was studied and compared to different polymers adhesion to treated and untreated aluminum surfaces by a series of shear strength tests. The chemical structural coherence was proven to take place between the modifier and PHFP film as the shear strength of the composite increased dramatically. This discovery opens new horizons for composite materials by showing the possibility of the direct synthesis customized surface modifiers for low surface free-energy fluorinated materials to be used as a constituent of different composites.

## Figures and Tables

**Table 1:** Anodized aluminum foil samples preparation.

№	Voltage, V	Background Electrolyte Composition	Time, Min
1	10	0.3 M sulfuric acid	15
2	60	0.3 M oxalic acid	15
3	60	0.3 M phosphoric acid	15
4	80	0.3 M phosphoric acid	15
5	100	0.3 M phosphoric acid	15
6	120	0.3 M phosphoric acid	15

## Data Availability

Not applicable.
